# Relationship between loneliness and self-efficacy: the mediating role of internet addiction and the moderating role of sleep quality

**DOI:** 10.3389/fpsyt.2026.1933890

**Published:** 2026-09-23

**Authors:** Xingwei Li, Yang Sun

**Affiliations:** 1 Baoding University of Technology, Baoding, China; 2 Jilin Agricultural University, Changchun, China

**Keywords:** adolescents, internet addiction, loneliness, self-efficacy, sleep quality

## Abstract

**Introduction:**

This study aimed to examine the association between loneliness and self-efficacy among adolescents and to investigate whether internet addiction mediated this association and whether sleep quality moderated the direct and indirect pathways. Although previous research has demonstrated significant associations between loneliness and both general and social self-efficacy, the behavioral mechanisms underlying these associations and the conditions under which their strength may vary remain insufficiently understood.

**Methods:**

A cross-sectional survey was conducted among 1,323 students in Grades 7–9 from two schools in Baotou, Inner Mongolia, China. Participants completed the UCLA Loneliness Scale, the General Self-Efficacy Scale, the Internet Addiction Diagnostic Questionnaire, and the Athens Insomnia Scale. A moderated mediation model was used to examine the mediating role of internet addiction and the moderating role of sleep quality in the association between loneliness and self-efficacy.

**Results:**

Loneliness was negatively associated with self-efficacy. Internet addiction partially mediated the association between loneliness and self-efficacy. In addition, sleep quality moderated the direct association between loneliness and self-efficacy and the first stage of the indirect pathway from loneliness to internet addiction. Specifically, the associations of loneliness with self-efficacy and internet addiction varied across different levels of sleep quality.

**Discussion:**

These findings provide a more comprehensive understanding of the mechanisms linking loneliness to adolescent self-efficacy by identifying internet addiction as an important behavioral mediator and sleep quality as a moderating factor. The findings highlight the importance of considering problematic Internet use and sleep quality when developing interventions aimed at promoting adolescents’ psychological adjustment, self-efficacy, and well-being.

## 1 Introduction

The mental health of adolescents is a key priority within the field of global public health. The World Health Organisation (WHO) notes that approximately one in seven adolescents (1) aged 10 to 19 worldwide is affected by mental health issues, accounting for around 15 per cent of the disease burden in this age group. It is worth noting that the majority of adolescents with mental health issues do not receive timely intervention; the consequences of this may extend into adulthood, thereby compromising their physical and mental well-being and their ability to adapt to society.

As an important period of rapid development in physical, psychological and social functions, adolescents experience significant emotional and social changes. In particular, the importance of peer relationships increases, while their self-awareness develops rapidly. Their demand for a sense of belonging and social connection also significantly increases (2, 3). However, when reality fails to meet these needs, individuals are likely to experience a sense of loneliness. A sense of loneliness refers to a negative emotional experience arising from an individual’ s failure to find fulfilment in their personal relationships (4). Recent studies have found that loneliness is one of the most important indicators reflecting mental health, and it is closely related to adverse reactions such as depression, anxiety, decreased self-esteem, and social adaptation (5, 6).

Compared to children and adults, the emotional development during adolescence is in a more sensitive period. Previous research has found that adolescents who experience a strong sense of loneliness tend to report higher levels of anxiety and depression, as well as lower levels of self-esteem (7). Thus, it is of great significance to explore the impact of loneliness on the positive psychological development of adolescents.

### 1.1 Loneliness and self-efficacy

As an early warning signal of mental health and behavioral problems in adolescents (8), loneliness has become more and more popular in recent years. In China, the loneliness level of adolescents is generally in a stable state, but shows a growth trend (6, 9). Long-term exposure to high levels of loneliness is associated with a range of negative emotional effects, such as feelings of isolation and helplessness, and may also have adverse effects on an individual’s immune and nervous system functions, thereby negatively impacting their physical and mental health (10, 11).

Self-efficacy is a key concept in Bandura’s social cognitive theory; it refers to an individual’s evaluation of their ability to organise and carry out a specific task (12), and is a subjective perception of the self (13). A high level of self-efficacy helps individuals to face challenges positively, enhances psychological resilience and promotes healthy development, whereas a low level of self-efficacy may lead to negative cognitions, avoidance behaviours and difficulties in adaptation (14).

According to social cognitive theory, self-efficacy arises not only from experiences of success, but is also influenced by the social environment and emotional states (15). Adolescence is a key period during which peer relationships develop rapidly; individuals spend a significant amount of time at school, and peer relationships and a sense of belonging play an important role in their social functioning (16). When an individual experiences a strong sense of loneliness in this social context, it often triggers negative emotional experiences, which in turn can lead to negative self-perception and negative self-evaluation (17). The negative emotional experiences accompanying loneliness can trigger negative self-experiences, and negative emotions can lead to adverse self-experiences, making individuals more likely to doubt themselves (18).

Studies have found that loneliness and general self-efficacy, social self-efficacy and emotion regulation self-efficacy exist significant negative correlation (19–22). Moreover, loneliness during adolescence not only leads to personal adaptation problems but also has adverse effects on the development of positive self-presentations and positive psychological qualities (23). Based on this, the Hypothesis 1 is proposed: Adolescent loneliness will be negatively associated with self-efficacy.

### 1.2 The mediating role of internet addiction

Emotional state is one of the important influencing factors of self-efficacy (24). When Bandura et al. explored the formation mechanism of self-efficacy, they found that an individual’s emotional experience would affect their judgment and evaluation of their own abilities, and thereby influence the development of self-efficacy (25). Current research has not explored the underlying mechanisms connecting emotion and self-efficacy in depth, focusing instead on the correlation between emotional states and self-efficacy. Therefore, it is necessary to further explore the underlying pathways through which negative emotions influence self-efficacy.

Loneliness, as a common negative emotion (26), may further affect self-efficacy through influencing an individual’s behavior. In the context of the widespread use of the internet, the internet has become an important channel for adolescents to meet their social needs (27). According to the affective processing model of negative reinforcement, when individuals experience the approach of negative emotions, they often turn to addictive behaviours in order to avoid or reduce the negative experience (28). For individuals who experience a strong sense of loneliness, the digital environment can provide immediate social feedback, social support and emotional compensation, thereby reducing their experience of loneliness to some degree (29).

Internet addiction refers to the use of the internet in an inappropriate or excessive manner, leading to damage to an individual’s psychological and social function (30, 31). Studies have found that loneliness is one of the important predictors of Internet addiction (32–34). A large number of studies from the adolescent to the college student population have shown that there is indeed a significant positive correlation between loneliness and internet addiction (35). Recent longitudinal evidence among primary and middle school students has also demonstrated close temporal associations between Internet addiction and anxiety symptoms, with anxiety showing a relatively stronger prospective association with subsequent Internet addiction symptoms (36). Individuals tend to rely on the internet to obtain emotional support and social connections in order to make up for the insufficient social needs in real life or the deficiencies in their own social skills, then increasing the risk of internet addiction (9, 37, 38).

Internet addiction is closely related to self-efficacy, and internet addicts often show lower levels of self-efficacy (39, 40). Among primary and secondary school students, there is a significant negative correlation between internet addiction and self-efficacy (41). According to the social cognitive theory, one’s own successful experiences are an important source of self-efficacy. Long-term exposure to the online world may reduce adolescents’ engagement with learning, social interaction and the accumulation of positive experiences in real life, thereby reducing their positive assessment of their own abilities and ultimately lowering their levels of self-efficacy.

In conclusion, Hypothesis 2 is proposed: Internet addiction plays a mediating role between loneliness and self-efficacy.

### 1.3 The regulatory role of sleep quality

Sleep quality is one of the important factors affecting the physical and mental health development of teenagers (42). Due to the heavy academic tasks and complex peer interaction pressure faced by middle school students, combined with irregular schedules, problems such as decreased sleep quality are easy to occur. Sleep quality is an individual’s subjective evaluation of their own sleep process, based on the individual’s objective sleep situation, mainly based on subjective feelings, and made as a judgment (43). Moreover, sleep quality issues not only have a significant impact on an individual’s mental health, but also have a close relationship with psychological behaviors such as internet addiction and self-efficacy (44, 45).

From the perspective of the protective factor model, protective factors can cushion the negative impact of risk factors on an individual’s development (46, 47). Sleep quality, as an important protective factor, may influence the process by which loneliness affects internet addiction and self-efficacy. Good sleep helps restore cognitive resources, enhance emotional regulation abilities, and improve an individual’s psychological resilience, while poor sleep quality weakens an individual’s ability to cope with stress and negative emotions (48).

For adolescents with high loneliness, poor sleep quality may further aggravate their negative emotional experience and make them more inclined to seek emotional compensation through the Internet, thus increasing the risk of Internet addiction. Previous studies have found that individuals with poor sleep quality are more likely to exhibit internet dependence and internet addiction tendencies (49). Therefore, sleep quality may regulate the relationship between loneliness and internet addiction.

Meanwhile, sleep quality may also affect the influence of loneliness on self-efficacy. According to the resource conservation theory, sleep helps individuals restore and accumulate psychological resources, and sufficient psychological resources are conducive to maintaining positive self-evaluation and perception of abilities (50). When individuals have better sleep quality, even if they experience a higher level of loneliness, they can maintain a higher level of self-efficacy through stronger emotional regulation ability and psychological resource reserve; conversely, when sleep quality is poor, the adverse effect of loneliness on an individual’s self-efficacy may be further enhanced.

Based on this, Hypothesis 3 is proposed: Sleep quality will moderate the association between loneliness and self-efficacy. Hypothesis 4: Sleep quality will moderate the association between loneliness and internet addiction.

In summary, current research has a number of shortcomings. Although studies to date have primarily focused on college students and older adults, there is a lack of research on adolescents, even though adolescence is an important period for the development of loneliness and self-efficacy. In addition, previous studies have mainly focused on the impact of loneliness on negative psychological outcomes such as depression and anxiety, while paying relatively less attention to positive psychological qualities like self-efficacy. Although existing empirical research has confirmed a clear correlation between loneliness and self-efficacy, the specific mechanism of their relationship has not been fully explained. In the context of the widespread use of the internet, the negative behavioral mechanisms caused by internet addiction and loneliness still need further exploration, and it is not clear which factors can buffer the adverse effects of loneliness on internet addiction and self-efficacy. Therefore, based on previous studies and theories, this research takes adolescents as the research subjects and proposes the following moderated mediation model to reveal whether loneliness was indirectly associated with self-efficacy through internet addiction and whether these associations varied across levels of sleep quality (**Figure 1**).

**Figure 1:**
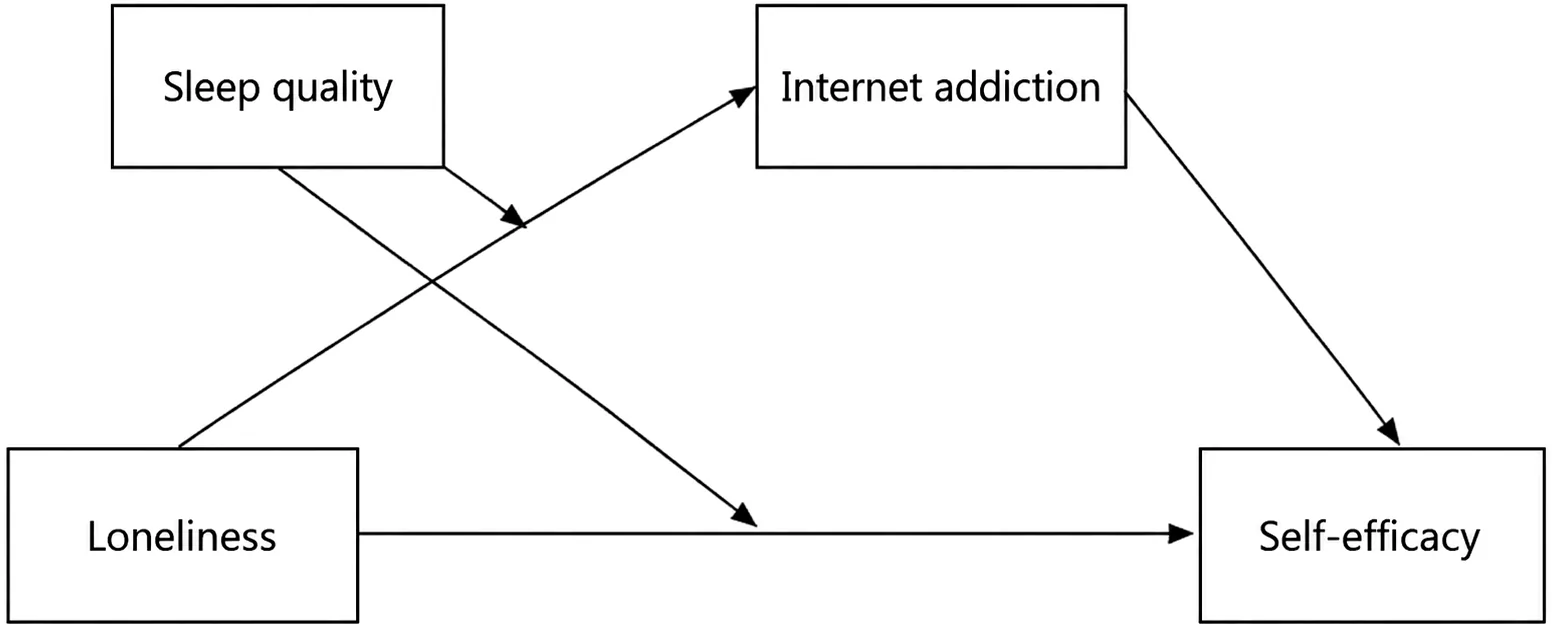
A model of the relationship between loneliness, self-efficacy, sleep quality and internet addiction.

## 2 Methods

### 2.1 Participants

A convenience sampling method was used to recruit participants from No. 24 Middle School and No. 28 Middle School in Baotou, Inner Mongolia, China. Within the two participating schools, all eligible students in Grades 7–9 were invited to participate as far as practicable in order to maximize the coverage of the target student population within each school. Participation was entirely voluntary. A total of 1,353 questionnaires were distributed, of which 1,323 were considered valid and included in the final analyses, yielding a valid response rate of 97.78%. The final sample consisted of 621 boys (46.94%) and 702 girls (53.06%), with 348 students (26.30%) in Grade 7, 386 (29.18%) in Grade 8, and 589 (44.52%) in Grade 9. Participants ranged in age from 11 to 17 years (M = 14.13, SD = 1.49).

### 2.2 Measures

#### 2.2.1 UCLA loneliness scale

Loneliness was assessed using Version 3 of the UCLA Loneliness Scale developed by Russell (4). The scale consists of 20 items, including 11 positively worded items assessing loneliness and 9 reverse-worded items assessing the absence of loneliness. All items were rated on a 4-point Likert scale ranging from 1 (“Never”) to 4 (“Always”). Higher total scores indicate greater levels of loneliness. In the present study, the scale demonstrated excellent internal consistency, with a Cronbach’s α coefficient of 0.909.

#### 2.2.2 Internet addiction diagnostic questionnaire

Internet Addiction was assessed using the eight-item Internet Addiction Diagnostic Questionnaire developed by Young. Each item was rated dichotomously (Yes = 1, No = 0). The eight items were summed to obtain a total score ranging from 0 to 8, with higher scores indicating greater severity of problematic Internet use. The total score was used in the subsequent statistical analyses. In the present study, the questionnaire demonstrated good internal consistency (Cronbach’s α = 0.820).

#### 2.2.3 Athens insomnia scale

Sleep quality was assessed using the Athens Insomnia Scale (AIS) developed by Soldatos et al. (51). The scale consists of eight items rated on a 4-point Likert scale ranging from 0 (“No sleep problems”) to 3 (“Severe sleep problems”). The original scoring direction of the AIS was retained, with higher total scores indicating poorer sleep quality (i.e., more severe sleep problems) and lower scores indicating better sleep quality. The scale demonstrated good internal consistency in the current study (Cronbach’s α = 0.886).

#### 2.2.4 General self-efficacy scale

Self-efficacy was measured using the Chinese version of the General Self-Efficacy Scale revised by Zhang Jianxin and colleagues (52). The scale consists of 10 items rated on a 4-point Likert scale ranging from 1 (“Not at all true”) to 4 (“Exactly true”). Higher scores indicate higher levels of general self-efficacy. In the present study, the Cronbach’s α coefficient was 0.898.

### 2.3 Procedure and statistical analysis

Prior to data collection, permission was obtained from the administrators of the participating schools. Participants completed the questionnaires after carefully reading and understanding the instructions, and sufficient time (at least 20 minutes) was provided to ensure thoughtful responses. After excluding invalid questionnaires, the data were entered into SPSS version 21.0 for statistical analyses. Descriptive statistics, Pearson correlation analyses, multiple regression analyses, mediation analyses, and moderation analyses were subsequently conducted.

### 2.4 Common method bias

To examine the potential influence of common method bias, Harman’s single-factor test was performed using exploratory factor analysis with an unrotated principal component solution. The results identified five factors with eigenvalues greater than 1, and the first factor accounted for 23.163% of the total variance, which was below the recommended threshold of 40%. These findings suggest that common method bias was not a serious concern in the present study.

## 3 Results and analysis

### 3.1 Descriptive statistics and correlation analysis of each variable

The average scores and correlation analysis of each variable in this study are shown in **Table 1**. Gender was significantly negatively correlated with age, internet addiction, and self-efficacy (*p* < 0.05), and was not correlated with other variables; grade was significantly negatively correlated with age (*p* < 0.01), and significantly positively correlated with self-efficacy (*p* < 0.01); age was significantly positively correlated with loneliness and sleep quality (*p* < 0.01), and significantly negatively correlated with self-efficacy (*p* < 0.01); loneliness and sleep quality were significantly negatively correlated with self-efficacy (*p* < 0.01); and were significantly positively correlated with internet addiction (*p* < 0.01); internet addiction was significantly negatively correlated with self-efficacy (*p* < 0.01). Loneliness and sleep quality were significantly positively correlated (*p* < 0.01), indicating that in the model, the independent variables and the moderating variables are independent of each other, thus there is a moderating effect (53).

**Table 1:** Descriptive statistics and Pearson correlation coefficients among loneliness, internet addiction, sleep quality, and self-efficacy.

Variable names	M±SD	1	2	3	4	5	6	7
1.Gender	--	--						
2.Grade	2.18±0.822	0.006	--					
3.Age	14.13±1.499	-0.065*	-0.204**	--				
4.Loneliness	38.57±11.353	-0.021	-0.037	0.127**	--			
5. Internet addiction	1.71±2.146	-0.059*	-0.029	0.041	0.249**	--		
6.Sleep quality	4.84±4.800	0.024	0.036	0.162**	0.476**	0.258**	--	
7. Self-efficacy	27.27±6.224	-0.027**	0.080**	-0.085**	-0.302**	-0.160**	-0.184**	--

*p<0.05, **p<0.01, ***p<0.001. The same notation applies to all subsequent tables.

### 3.2 Mediation analysis

After controlling for gender, grade, and age, the indirect association between loneliness and self-efficacy through internet addiction was examined. The results are presented in **Table 2**. Loneliness was significantly and negatively associated with self-efficacy (*β* = −0.296, *p* <.001) and significantly and positively associated with internet addiction (*β* = 0.247, *p* <.001). When loneliness and internet addiction were simultaneously entered into the regression model, loneliness remained significantly and negatively associated with self-efficacy (*β* = −0.273, *p* <.001), while internet addiction was also significantly and negatively associated with self-efficacy (*β* = −0.094, *p* <.001).

**Table 2:** Regression analyses examining the mediating effect of internet addiction.

Regression model		Model fit			Regression coefficients			
Outcome variable	Predictor	*R*	*R²*	*F*	*β*	Lower CI	Upper CI	*t*
Self-efficacy	Gender	0.321	0.103	37.976	-0.162	-0.265	-0.059	-3.095**
	Grade				0.074	0.010	0.138	2.285*
	Age				-0.027	-0.062	0.009	-1.483
	Loneliness				-0.296	-0.348	-0.244	-11.257***
Internet addiction	Gender	0.256	0.065	23.010	-0.106	-0.211	-0.001	-1.989*
	Grade				-0.023	-0.088	0.042	-0.691
	Age				0.002	-0.034	0.038	0.084
	Loneliness				0.247	0.194	0.299	9.199***
Self-efficacy	Gender	0.334	0.112	33.086	-0.172	-0.275	-0.069	-3.295**
	Grade				0.072	0.009	0.135	2.228*
	Age				-0.027	-0.062	0.009	-1.481
	Internet addiction				-0.094	-0.147	-0.041	-3.498***
	Loneliness				-0.273	-0.326	-0.219	-10.099***

All variables included in the regression models were standardized prior to analysis. The lower and upper limits of the 95% confidence interval (CI) are reported as the Lower CI and Upper CI, respectively. *p < 0.05, **p < 0.01, and ***p < 0.001.

Further bootstrap analyses indicated that Internet addiction significantly mediated the relationship between loneliness and self-efficacy. The indirect effect was −0.023, with a 95% bootstrap confidence interval (CI) ranging from −0.040 to −0.007, excluding zero. The indirect effect accounted for 7.84% of the total effect.

### 3.3 Testing the moderated mediation model

SPSS Process Model 8 was used to test the moderated mediation model. After controlling for gender, grade, and age, the moderating effect of sleep quality was examined within the mediation model. The results are presented in **Table 3**. As shown in Model 1, loneliness significantly predicted self-efficacy, and the interaction between loneliness and sleep quality was significantly associated with self-efficacy. These findings indicate that sleep quality moderated the direct association between loneliness and self-efficacy, while Internet addiction partially mediated this relationship. Model 2 showed that the interaction between loneliness and sleep quality significantly were significantly associated with Internet addiction, indicating that sleep quality moderated the first stage of the mediation process (i.e., the association between loneliness and Internet addiction). As shown in Model 3, the interaction between loneliness and sleep quality was significantly associated with self-efficacy, suggesting that the moderating effect of sleep quality on the direct pathway persisted after including the mediator. In contrast, the interaction between Internet addiction and sleep quality did not significantly associated with self-efficacy, indicating that sleep quality did not moderate the second stage of the mediation process (i.e., the association between Internet addiction and self-efficacy). Taken together, these findings suggest that sleep quality moderated both the direct effect of loneliness on self-efficacy and the first stage of the indirect pathway through Internet addiction.

**Table 3:** Regression analyses for the moderated mediation model.

Outcome variable	Model 2 (Internet addiction)				Model 3 (Self-efficacy)			
	*β*	*t*	Lower CI	Upper CI	*β*	*t*	Lower CI	Upper CI
Loneliness	0.163	5.428***	0.124	0.222	-0.268	-9.002***	-0.327	-0.210
Sleep quality	0.212	6.531***	0.148	0.275	-0.087	-2.688**	-0.151	-0.025
Loneliness × Sleep quality	-0.046	-1.965*	-0.093	-0.0001	0.108	4.646***	-0.136	-0.029
Internet addiction					-0.082	-3.041**	0.062	0.154
*R²*	0.945				0.127			
*F*	22.958				27.24			

All variables included in the regression models were standardized prior to analysis. The lower and upper limits of the 95% confidence interval (CI) are reported as the Lower CI and Upper CI, respectivel. *p < 0.05, **p < 0.01, and ***p < 0.001.

To further illustrate the moderating effect of sleep quality on the relationship between loneliness and self-efficacy, simple slopes analyses were conducted by categorizing sleep quality into high (+1 SD) and low (−1 SD) levels. The corresponding simple slope plots and regression results are presented in **Figure 2** and **Table 4**, respectively.

**Figure 2:**
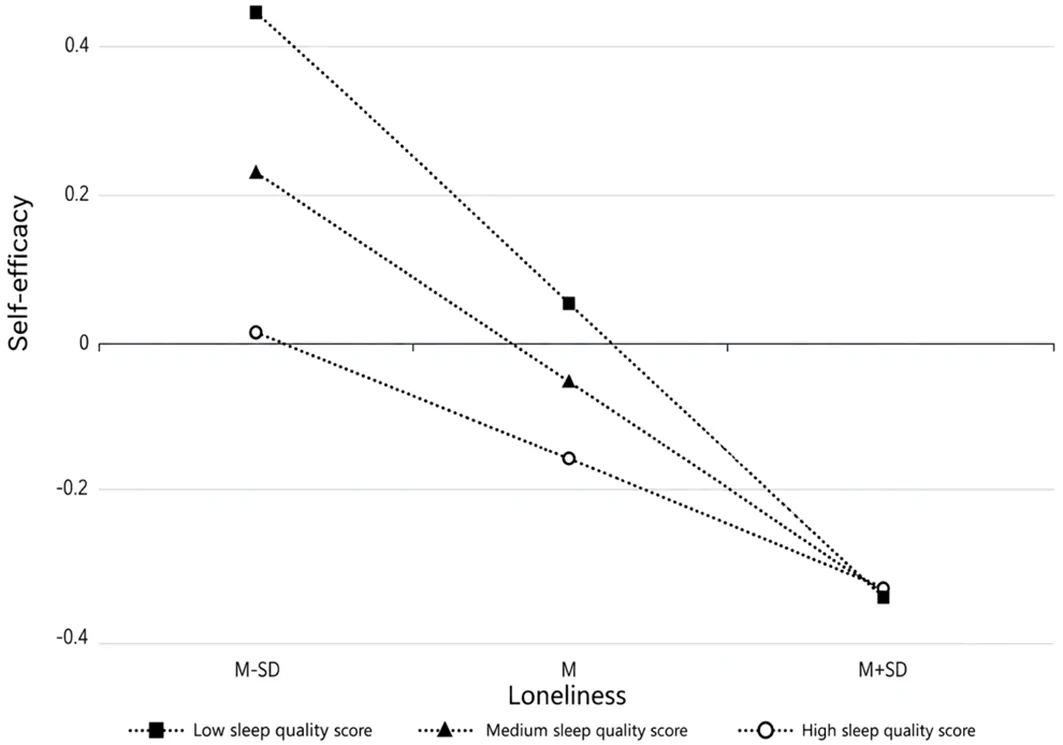
The moderating effect of sleep quality on the relationship between loneliness and self-efficacy.

**Table 4:** Conditional effects of loneliness on self-efficacy at different levels of sleep quality.

Level of sleep quality	Effect	SE	Lower CI	Upper CI	*t*
-1 (M-SD)	-0.376	0.039	-0.452	-0.299	-9.665***
0 (M)	-0.268	0.029	-0.327	-0.210	-9.002***
1 (M+SD)	-0.160	0.037	-0.232	-0.088	-4.374***

Lower CI and Upper CI represent the lower and upper bounds of the 95% confidence interval, respectively. ***p < 0.001.

The results showed that loneliness was significantly negatively associated with self-efficacy at both high (β_simple = −0.16, *p* <.001) and low (β_simple = −0.38, *p* <.001) levels of sleep quality. Notably, the negative association between loneliness and self-efficacy was weaker among individuals with higher sleep quality score.

The moderating effect of sleep quality on the association between loneliness and Internet addiction was further examined using simple slopes analyses, with the results presented in **Figure 3** and **Table 5**. Loneliness was significantly positively associated with Internet addiction at both high (β_simple = 0.12, *p* <.01) and low (β_simple = 0.21, *p* <.001) levels of sleep quality. However, the positive association was weaker among individuals with higher sleep quality score.

**Figure 3:**
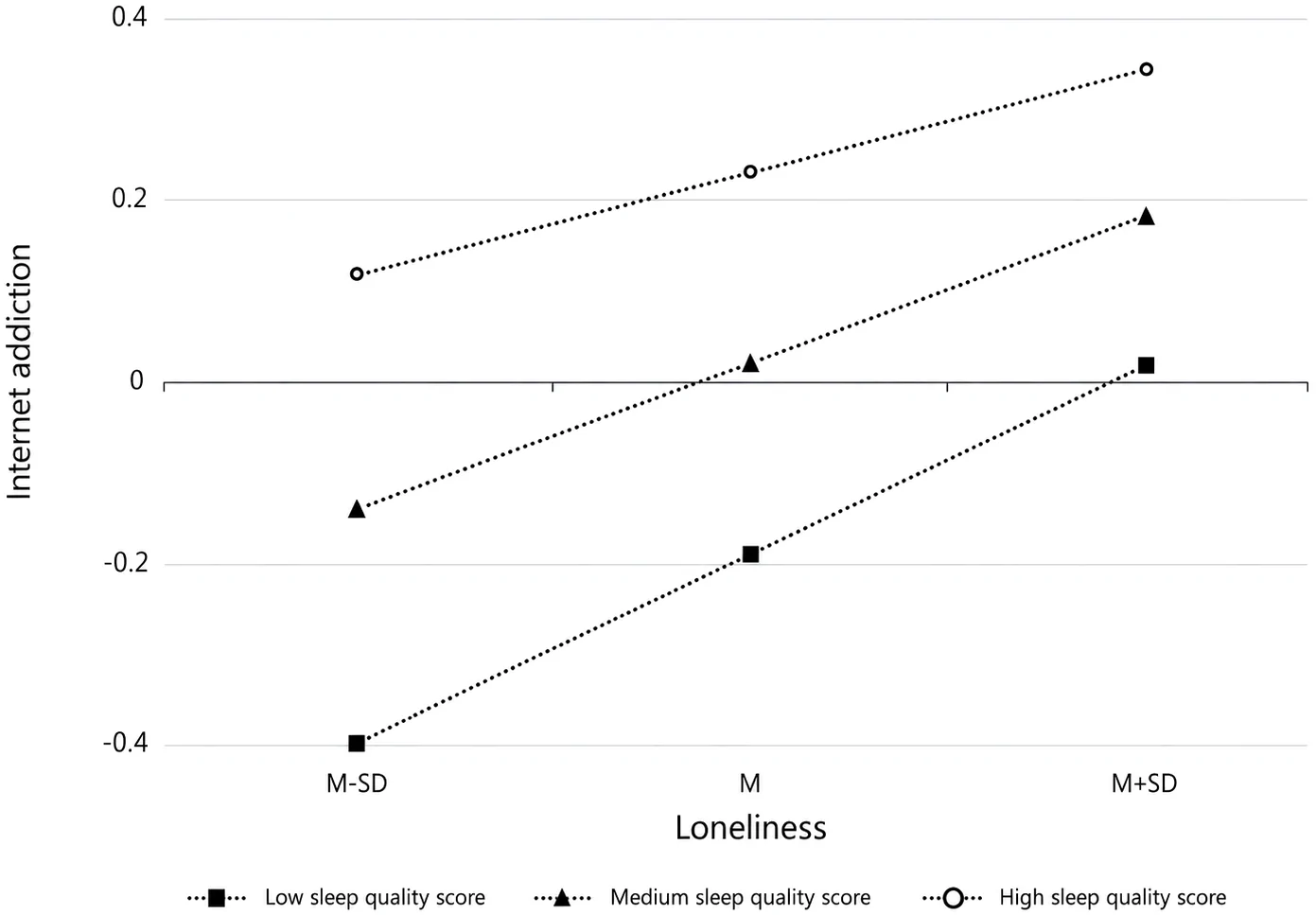
The moderating effect of sleep quality on the relationship between loneliness and internet addiction.

**Table 5:** Conditional effects of loneliness on internet addiction at different levels of sleep quality.

Levels of sleep quality	Effect	SE	Lower CI	Upper CI	*t*
-1 (M-SD)	0.209	0.039	0.132	0.286	5.338***
0 (M)	0.163	0.030	0.104	0.222	5.428***
1 (M+SD)	0.116	0.037	0.044	0.189	3.133**

Lower CI and Upper CI represent the lower and upper bounds of the 95% confidence interval, respectively. **p < 0.01, and ***p < 0.001.

## 4 Discussion

This study examined the associations among adolescent loneliness, self-efficacy, internet addiction, and sleep quality. The results demonstrated that loneliness, self-efficacy, sleep quality and internet addiction constitute a moderated regulatory model with mediating effects. Loneliness has a direct impact on self-efficacy, and loneliness can also indirectly affect self-efficacy through internet addiction. These relationships vary slightly among individuals with different sleep quality.

### 4.1 Loneliness and self-efficacy

The results of this study demonstrate that loneliness was significantly and negatively associated with self-efficacyself-efficacy: the higher the level of loneliness, the lower the level of self-efficacy, and conversely, the lower the level of loneliness, the higher the level of self-efficacy. Hypothesis 1 is supported by these findings, which are consistent with previous research (21) and further highlight that loneliness is one of the key risk factors affecting adolescents’ mental health.

In addition, recent studies have found a close connection between loneliness and low self-esteem (5), and low self-esteem can lead to arise self-doubt. In Bandura’s theory of self-efficacy, the influence of emotions on self-efficacy is emphasized. The emotional state of an individual directly affects their self-efficacy (25). Both self-esteem and self-efficacy are important components of an individual’s positive self-system. When an individual is in a state of loneliness for a long time, their positive self-evaluation system may be damaged, thereby further reducing the level of self-efficacy.

### 4.2 The mediating role of internet addiction

The results of this study suggest that internet addiction partially mediates the relationship between loneliness and self-efficacy; loneliness can reduce self-efficacy both directly and indirectly via internet addiction. Hypothesis 2 is supported.

Firstly, this study found that loneliness was significantly and positively associated with internet addiction. This finding is consistent with the Compensatory Internet Use Theory, which suggests that when individuals experience stress, setbacks or unsatisfied social needs in real life, they tend to turn to the internet to cope with the negative effects of these experiences (54). Adolescence is an important stage for the development of internet addiction (55, 56). For adolescents with high loneliness, online environment can provide immediate feedback, emotional support and virtual social connection, so it is easier to become an important way to meet their belongingness needs. Previous studies have found that individuals with higher levels of loneliness are more likely to use online social interaction as a substitute for real social interaction and try to make up for the social deficiencies in reality through the internet (57). Therefore, the stronger the loneliness, the higher the risk of internet addiction.

Secondly, this study found that internet addiction can significantly and indirectly reduce self-efficacy. According to the social cognitive theory, one of the important sources of self-efficacy is the individual’s successful experiences in real life and the positive evaluation of their own abilities. When individuals rely too heavily on the internet to satisfy their psychological needs, their engagement with real life may decrease accordingly, consequently impacting their ability to accumulate experiences of success and develop social adaptability. As the positive feedback in the real environment decreases, individuals are more likely to doubt their own abilities, thereby reducing the level of self-efficacy. The results of this study are consistent with previous research findings. Yao et al. (39) pointed out that individuals with Internet addiction often show characteristics such as isolation, sensitivity, emotional instability and low self-efficacy. A large number of empirical studies have also shown that there is a significant negative correlation between internet addiction and self-efficacy, that is, the higher the level of internet addiction, the lower the self-efficacy (58). Moreover, internet addiction also has negative effects on different dimensions such as academic self-efficacy, social self-efficacy, and emotional regulation self-efficacy (59).

### 4.3 The regulatory role of sleep quality

With regard to self-efficacy, loneliness was negatively associated with self-efficacy at all levels of sleep quality, but this negative association was stronger among adolescents with lower sleep quality scores (i.e., better sleep quality) and weaker among those with higher sleep quality scores (i.e., poorer sleep quality). Inspection of the interaction plot further suggests that adolescents with better sleep quality reported relatively higher self-efficacy when loneliness was low, whereas the difference between sleep groups gradually diminished as loneliness increased.

One possible interpretation can be derived from conservation of resources theory. This theory proposes that individuals with insufficient psychological and physiological resources are more vulnerable to adverse psychological functioning (60). Sleep plays an important role in restoring energy and maintaining psychological functioning (61), whereas poor sleep is often accompanied by fatigue and impaired cognitive functioning (62). Fatigue has also been associated with lower self-efficacy in previous research (63). Consistent with this perspective, previous studies have reported positive associations between sleep quality and self-efficacy (64, 65). Accordingly, adolescents with better sleep may have more favorable cognitive and psychological resources with which to maintain positive evaluations of their own competence when loneliness is relatively low. However, when loneliness becomes elevated, the adverse psychological correlates of persistent social disconnection may become sufficiently salient that the self-efficacy advantage associated with better sleep is reduced. In this sense, the interaction observed here may reflect a convergence pattern at higher levels of loneliness rather than a conventional buffering effect of sleep quality.

With regard to internet addiction, loneliness was positively associated with internet addiction at all levels of sleep quality, but this positive association was weaker among adolescents with higher sleep quality scores (i.e., poorer sleep quality). At the same time, inspection of the interaction plot indicates that adolescents with poorer sleep quality consistently reported relatively higher levels of Internet addiction across different levels of loneliness. This pattern suggests that poor sleep quality may be associated with a generally elevated level of Internet addiction, whereas loneliness may represent a relatively stronger correlate of Internet addiction among adolescents with better sleep quality.

Previous research has consistently identified a close association between sleep problems and problematic Internet use. For example, poorer sleep quality has been associated with Internet dependence and Internet addiction tendencies (49), and associations between Internet addiction and impaired sleep quality have also been reported in student populations (44, 45). These findings provide a possible context for interpreting the present interaction. Adolescents with more severe sleep problems may already experience a relatively high level of problematic Internet use even when loneliness is low. Consequently, loneliness may account for a smaller proportion of individual differences in Internet addiction within this group. By contrast, among adolescents with better sleep quality, Internet addiction is relatively low when loneliness is low, and the increase associated with higher loneliness is therefore more pronounced.

Thus, the weaker loneliness–internet addiction association among adolescents with poorer sleep quality should not be interpreted as indicating that poor sleep has a protective function. Rather, it may reflect an elevated baseline level of Internet addiction associated with sleep problems, which reduces the relative contribution of loneliness to individual differences in Internet addiction. This pattern further suggests that problematic Internet use may be associated with multiple co-occurring psychological and behavioral difficulties rather than with loneliness alone.

Overall, the findings indicate that sleep quality constitutes an important contextual factor associated with variation in the strength of the relationships among loneliness, self-efficacy, and Internet addiction. However, the observed patterns are more consistent with convergence and elevated-baseline interpretations than with a simple protective-buffering model. Further longitudinal studies are required to clarify the temporal ordering and developmental processes underlying these associations.

### 4.4 Limitations

Although this study has highlighted the relationships among loneliness, self-efficacy, problematic Internet use, and sleep quality, several limitations should be acknowledged. First, all variables were assessed using self-report questionnaires administered at the same time. Although Harman’s single-factor test did not indicate a serious common method problem, this test alone cannot rule out common method bias. The use of a single measurement method and source may also introduce social desirability and other response biases. Future studies could incorporate multiple informants, behavioral indicators, or objective measures to reduce these potential biases. Secondly, participants were recruited through convenience sampling from only two schools in Baotou, Inner Mongolia. Although efforts were made to maximize student coverage within the participating schools, the sample cannot be considered representative of adolescents in other regions or educational contexts. Secondary school students from different regions and cultural backgrounds may show differences in their psychological development or behaviour; in future, samples could be drawn from a range of regions and cultural contexts to further validate the generalisability of this study’s findings. Third, although the mediating effect of internet addiction on the relationship between loneliness and self-efficacy was statistically significant, the indirect effect accounted for only 7.84% of the total effect. This relatively small proportion indicates that internet addiction may represent only one secondary pathway linking loneliness to self-efficacy, whereas the direct effect remains predominant. Therefore, the mediating role of internet addiction should be interpreted with appropriate caution. Other psychological, social, or behavioral mechanisms not examined in the present study may also contribute to this relationship. Future research could investigate additional mediating factors to provide a more comprehensive understanding of the mechanisms through which loneliness is associated with self-efficacy.

Additionally, this study verified the moderating effect of sleep quality, but did not directly measure the specific psychological mechanism through which it exerts its protective effect. For instance, sleep quality may play a protective role by influencing emotional regulation ability, cognitive resource recovery or psychological resilience, among other pathways. Future research can further incorporate relevant variables to deeply explore the specific mechanism of how sleep quality affects loneliness. Finally, as this study used a cross-sectional design, although the results support the proposed model of the relationship between loneliness, internet addiction, self-efficacy and sleep quality, they do not fully validate the causal relationships between the variables. Future research could adopt a longitudinal or experimental design to further examine the dynamic developmental processes and causal mechanisms between these variables.

## 5 Conclusions

(1) Adolescent loneliness was significantly and negatively associated with self-efficacy; (2) loneliness showed a significant indirect association with self-efficacy through internet addiction; (3) sleep quality moderated the association between loneliness and self-efficacy; and (4) sleep quality moderated the association between loneliness and internet addiction.

## Data Availability

The raw data supporting the conclusions of this article will be made available by the authors, without undue reservation.
